# Successful ventricular tachycardia radioablation in a patient with previous chemical pleurodesis: A case report

**DOI:** 10.3389/fcvm.2022.937090

**Published:** 2022-07-18

**Authors:** Chiara Pavone, Roberto Scacciavillani, Maria Lucia Narducci, Francesco Cellini, Gemma Pelargonio, Gianluigi Bencardino, Francesco Perna, Francesco Spera, Gaetano Pinnacchio, Tommaso Sanna, Vincenzo Valentini, Filippo Crea

**Affiliations:** ^1^Department of Cardiovascular Sciences, Agostino Gemelli University Polyclinic (Istituto di Ricovero e Cura a Carattere Scientifico), Rome, Italy; ^2^Unit of Oncological Radiotherapy, Department of Diagnostic Imaging, Oncological Radiotherapy and Hematology, Agostino Gemelli University Polyclinic (Istituto di Ricovero e Cura a Carattere Scientifico), Rome, Italy

**Keywords:** ventricular tachycardia, stereotactic arrhythmia radioablation, chemical pleurodesis, case report, epicardial ventricular tachycardia ablation

## Abstract

**Introduction:**

Stereotactic arrhythmia radioablation (STAR) is a novel technique for the ablation of ventricular tachycardia in patients with contraindications to standard procedures, i.e., radiofrequency ablation.

**Case presentation:**

We report the case of a 73-year-old man with non-ischemic dilated cardiomyopathy and recurrent VT episodes. Electroanatomic mapping showed VT prevalently of epicardial origin, but direct epicardial access through subxyphoid puncture could not be performed due to pleuropericardial adhesions from a past history of chemical pleurodesis. STAR was performed, with no VT recurrence at 6 months follow-up.

**Conclusions:**

Previous experiences with STAR have demonstrated its importance in the management of patients with refractory VT in whom other ablation strategies were not successful. Our case report highlights the use of STAR as a second choice in a patient with an unfavorable VT anatomical location and technical limitations to an optimal radiofrequency ablation. Moreover, it confirms STAR's effectiveness in the ablation of complex transmural lesions, which are more often associated with non-ischemic structural heart disease.

## Introduction

Stereotactic arrhythmia radioablation (STAR) is a technique in which a single high dose of focused stereotactic radiation is employed for the ablation of cardiac arrhythmias (1). So far, STAR's main field of application has been in the ablation of recurrent monomorphic ventricular tachycardia (VT) (2).

The current gold standard for recurrent VT management is represented by percutaneous radiofrequency ablation (RFA) (3). The conventional procedure involves VT mapping and ablation through an endocardial approach. However, the electroanatomical substrate responsible for VT formation is not always located endocardially, so an epicardial approach is sometimes necessary in order to effectively ablate the arrhythmia.

Since a first case study published by Cuculich et al. in 2017 (4), STAR has been proposed as an alternative to percutaneous RFA in those who cannot undergo such procedure due to patient contraindications or limitations to the procedure itself.

We present the case of effective VT ablation through STAR in a 73 year-old man with recurrent VT of epicardial origin in whom a direct epicardial ablation could not be performed because of a past history of chemical pleurodesis.

## Case presentation

**Figure 1:**
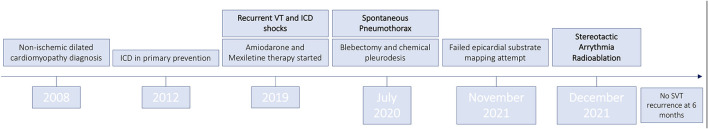
Patient clinical history timeline.

A 73-year-old man suffering from non-ischemic dilated cardiomyopathy in NYHA class I was admitted to our hospital because of recurrent VT episodes. Figure 1 summarizes the patient's clinical history.

Cardiovascular risk factors included: former smoking habit, dyslipidemia and a long-term history of systemic arterial hypertension. Noteworthy comorbidities included chronic obstructive pulmonary disease (COPD) and obstructive sleep apnea syndrome (OSAS) treated with long-term oxygen therapy and nocturnal continuous positive airway pressure (CPAP). In July 2020 he suffered an episode of spontaneous pneumothorax (PNX), which was successfully treated with blebectomy and chemical pleurodesis.

The underlying cause of the patient's cardiomyopathy had been studied throughout the years by performing multiple coronary angiographies, which showed no evidence of significant coronary artery stenoses. In 2012 he was implanted with a single-chamber implantable cardioverter-defibrillator (ICD) in primary prevention. He also underwent successful atrial fibrillation catheter ablation and atrial flutter catheter ablation.

About 1 year before hospitalization, the patient started experiencing frequent ICD shocks (often during the night), which severely impacted his quality of life. In May 2020, device interrogation showed recurrent VT episodes, either sustained or interrupted by antitachycardia pacing (ATP) or direct-current (DC) shock, therefore optimized anti-arrhythmic therapy with Amiodarone 200 mg twice daily and Mexiletine 200 mg three times a day was started. In April 2021, an electrophysiological study (EPs) showed induction of multiple VTs of different morphologies. All induced VTs caused hemodynamic instability.

The patient was admitted to our Cardiology department in November 2021 to undergo VT ablation. During the 2 months before admission, device interrogation showed that the patient experienced 4 episodes of VT recurrence, all ended by DC shock.

A baseline echocardiogram showed left ventricle (LV) hypertrophy (IVS: 17 mm) and dilation (LVEDVi 76 ml/mq) and a moderately reduced ejection fraction (50%), with inferior basal wall hypokinesia. Cardiac Magnetic Resonance imaging (MRI) was not performed due to the presence of a non MRI-conditional device.

**Figure 2:**
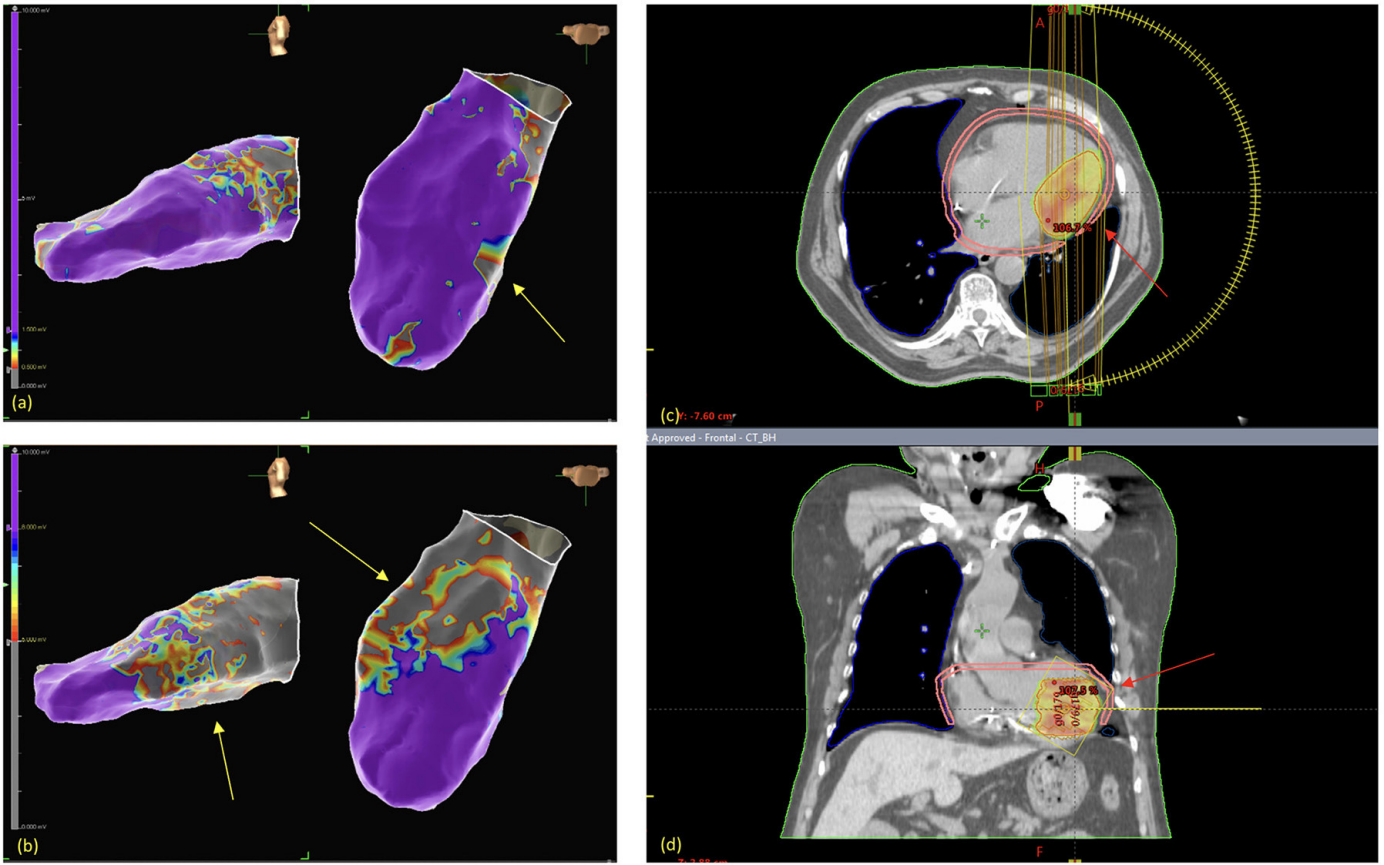
Left ventricular (LV) electroanatomic mappings (EAM) show normal voltage areas (in purple), as opposed to low voltage areas (in grey and other colors, representing LV scar area). Bipolar EAM **(a)** shows inferior-mid LV low voltage areas (yellow arrows), while unipolar EAM **(b)** shows a larger scar area on the inferior-lateral LV. STAR treatment planning shows the planning target volume (red arrows) in the axial **(c)** and coronal **(d)** planes.

3D Electroanatomic mapping (EAM) of the left ventricle was carried out with the Abbott EnSite system by retroaortic approach. The bipolar map showed low voltage areas (bipolar voltage 0.5–1.5 mV) in the inferior mid LV (total area: 1.4 cm^2^), while the unipolar map showed a more extended low voltage area (unipolar voltage 5.5–8 mV) in the inferolateral LV (total area: 2.8 cm^2^). Two VTs were induced: the first one (VT1) showed a RBBB morphology, with a cycle length (CL) of 345 s (174 bpm); the second (VT2) showed a CL of 322 s (186 bpm). Figures 2A,B respectively show Bipolar and Unipolar EAMs of the patient's left ventricle.

Since the arrhythmia was not hemodynamically tolerated, endocardial substrate mapping was attempted but turned out to be unsuccessful because of VT2's persistent inducibility at programmed ventricular stimulation. Moreover, various ECG features pointed toward VT of epicardial origin (MDI 0.55) (5). Epicardial approach through percutaneous subxyphoid puncture was therefore attempted. Three attempts from three different electrophysiologists were made, all failing due to the impossibility to advance the guidewire in the pericardial space. Considering the patient's past history of chemical pleurodesis and the related pleuro-pericarditic reaction, no further direct epicardial access attempts were made. STAR was therefore proposed to the patient and informed consent was given.

## STAR: Planning and delivery

### Treatment simulation

The patient was positioned in the supine position with arms raised above the head. The patient was immobilized with vac-lok cushions (CIVCO Vac-LokTM Cushions). Planning CT (GE, Optima CT580 W, HiSpeed DX/I Spiral) without contrast agent was acquired. Multiple CT acquisition has been performed for simulation: (i) Free breathing CT; (ii) Four dimensional (4D)CT; (iii) Deep inspiration Breath hold CT (DIBH). That enabled respiratory gated delivery approach.

### Target definition and delineation

Average CT scan, computed by the 4DCT, was selected for target (in term of Internal Target Volume -ITV-) and Organ at Risk (OAR) delineation, with a slice thickness of 2.5 mm. Target area (TA) delineation was obtained by merging electroanatomic mapping images with the planning CT scan; merging was applied through a screen-to screen qualitative approach, the anatomical areas of the CT simulation corresponding to the EAM were independently outlined by the radiation oncologist and eventually double-checked by the radiation oncologist with one of the electrophysiologists having performed the EAM. A margin of 3 mm was added to obtain Planning Target Volume (PTV).

### Treatment planning

STAR was planned in one fraction using the TrueBeam Edge Linac. The prescribed dose was 25 Gy to the 80% isodose. We used the TrueBeam Edge Linac (Varian Medical Systems, Palo Alto, CA) with 6MV flattening filter free photons and dose calculation algorithm Acuros (Eclipse Version 15.6.04 Varian Medical Systems, Palo Alto, CA). Volumetric Modulated Arc Therapy (VMAT) technique was used with three partial arcs. We did apply the consraints indicated by the AAPM TG101 report (6). All constraints were within the tolerance based on the AAPM TG101 report, except for the pericardia that has been optimized, decreasing the dose as low as reasonably achievable, without compromising target coverage.

### Treatment delivery

The patient was treated without the use of either sedation or anesthesia. Image guidance was performed by both volumetric imaging (by Cone Beam CT -CBCT-) before each PA delivery for positioning verification and correction, and by optical surface monitor system (OSMS) for continuous positioning intrafraction tracking and delivery triggering.

The patient was aligned at the isocenter, and 3-dimensional volumetric image guidance through CBCT was acquired. Three- dimensional alignment was performed progressively matching for alignment against the reference images of the treatment plan: bone structures, then organs (e.g., lungs), whole heart, then finally the Clinical Target Volume (CTV). For OSMS: reference image was firstly acquired, defining the tracking Region of Interest (ROI).

Once OSMS was set-up, the first PA was delivered. A CBCT was acquired before each PA. OSMS recorded patient positioning and triggered the beam delivery whenever out of tolerance during the entire delivery procedure. A total dose of 25 Gy in single fraction was delivered. Pre-treatment patient setup was performed in 15 min, including the acquisition of the OSMS reference imaging.

No interruption due to patient related factors was necessary, in particular no ventricular arrhythmia occurred during the intervention. Implantable Cardioverter Defibrillator (ICD) function was normal and cardiac enzymes remained stable after the radiation treatment. Figures 2C,D show radiation therapy treatment planning.

## Clinical outcomes

Following the procedure, the patient presented no VT recurrence or acute complications during hospital stay. Post-procedural echocardiography documented the presence of a mild circumferential pericardial effusion (maximum 5 mm) with no hemodynamic impact. The patient was discharged 3 days after the procedure with optimized antiarrhythmic therapy, as described above.

At 3 months follow up, the patient presented no recurring VT episodes on device interrogation. He also reported a significant improvement in quality of life. In light of such findings, Amiodarone dosage was reduced to 200 mg once daily. Follow up echocardiography showed no significant ejection fraction reduction (the patient remained in moderately reduced ejection fraction heart failure, EF: 45%) and complete resolution of the pericardial effusion. 5 months following the procedure, the patient experienced an episode of palpitations. Device interrogation showed a single non-sustained VT, in the absence of other ventricular arrhythmias. On echocardiographic reevaluation, the ejection fraction remained stable (FE: 47%).

No STAR-related side effects were recorded, according to the CTCAE classification v 6.0 (7).

## Discussion

Patients with structural heart disease (e.g., ischemic and non-ischemic dilated cardiomyopathy) often suffer from recurrent VT, which greatly affects their prognosis and quality of life, and is generally managed through medical therapy (Class III antiarrhythmic drugs), RFA (the gold standard) and ICD implantation (8).

The use of RFA can be limited by intrinsic technical aspects of the intervention, such as difficult myocardial scar anatomical location, intraprocedural complications, and patient contraindications to the procedure. First and foremost, the invasive nature of percutaneous RFA may limit its use in frail subjects, therefore a completely non-invasive ablation strategy could be a life-saving alternative in this population.

Myocardial scars responsible for reentry mechanisms behind VT recurrence can sometimes be difficult to reach through standard ablation procedures. An example is represented by transmural scars mainly localized on the epicardial surface, which are more commonly reported in patients with non-ischemic dilated cardiomyopathy (9). In such cases, standard endocardial ablation can be complemented by the use of epicardial mapping and ablation through surrounding anatomical structures (e.g., coronary veins, coronary cusps) or through percutaneous subxyphoid pericardial puncture, which allows direct access to the epicardium (10). This procedure carries a series of risks and complications, which include RV puncture and pericardial bleeding, damage to coronary arteries and injury to surrounding structures (11). In addition, patients may present preexisting conditions that make the direct epicardial access unfeasible. Examples include tissue adhesions due to previous chest surgery or an anatomically unfavorable conformation of the thorax.

Chemical pleurodesis involves the administration of sclerosing agents to prevent pneumothorax recurrence. These chemical irritants induce a cytokine-induced inflammatory reaction and consequent fibroblast proliferation, which result in pleural space obliteration (12). In our case report, direct epicardial access through subxyphoid pericardial puncture was hindered by pleurodesis-induced fibrosis, therefore complete transmural VT ablation could not be performed. Following STAR treatment, our patient experienced an immediate significant reduction in VT burden. At 6 months follow up, there were no sustained VT recurrences nor STAR-related side effects, with a patient-reported significant improvement in quality of life.

According to the literature, an acute reduction in ventricular arrhythmia burden is seen in most patients within few days or weeks from STAR treatment. However, sustained VT/VF recurrence was reported in 75% of the studied population, mostly within the first 6 months (13). In our case report, no sustained VT episodes were registered in the first 6 months following treatment. Longer term durability of the effects of STAR in our patient are unknown.

Few mechanisms underlying the effects of radiation in VT ablation have been proposed. Preclinical studies showed inflammatory cell infiltration and fibrin deposition in radiation-exposed myocardial areas (14). Accordingly, a study by Kiani et al. (15) demonstrated the presence of subendocardial fibrosis and signs of acute myocardial injury in explanted hearts from patients who received orthoptic heart transplantation. Nonetheless, radiation-induced fibrosis is typically seen after a blanking period of a few weeks and cannot explain the reduction in VT burden seen in our patient immediately following the procedure.

Anticipated STAR effects may be explained by a preclinical study by Zhang et al. (16) that showed how radiotherapy also induces myocardial electrical conduction reprogramming through increased expression of sodium channels (Nav1.5), upregulation of Connexin 43 (involved in gap junction coupling) and Notch signaling activation. Their findings also elucidate a possible reason behind STAR's effectiveness on transmural scars despite the fact that the standard 25 Gy radiation dose doesn't create transmural myocardial lesions in animal models (17).

This case report highlights the importance STAR is gaining in every day's clinical practice. STAR is mainly regarded as the last option when all other possibilities (pharmacological and interventional) fail. Notably, its main field of application has been in, but not limited to, elderly end-stage heart failure patients unable to tolerate a long and complex VT ablation procedure. Our patient was a 73 years old man, in NYHA class I and with a moderately reduced ejection fraction and recurrent episodes of VT. After STAR, the subject experienced no more VT episodes and went back to his normal, active life. Our case report therefore confirms previous findings that STAR is not only a last resort treatment option in advanced structural heart disease patients, but that it can be a safe and effective alternative for the management of lower NYHA class patients whose quality of life is greatly affected by VT recurrence.

To the best of our knowledge this is the first case of STAR of a VT in a patient with difficulty in obtaining epicardial access due to previous chemical pleurodesis.

In the next future, thanks to the continuous refinement of the technique and with the expansion of its availability, STAR has the possibility to become one of the weapons in electrophysiologist's arsenal rather than a last resort tool.

## Data availability statement

The original contributions presented in the study are included in the article/supplementary material, further inquiries can be directed to the corresponding author/s.

## Ethics statement

Written informed consent was obtained from the individual(s) for the publication of any potentially identifiable images or data included in this article.

## Author contributions

All authors listed have made a substantial, direct, and intellectual contribution to the work and approved it for publication.

## Conflict of interest

The authors declare that the research was conducted in the absence of any commercial or financial relationships that could be construed as a potential conflict of interest.

## Publisher's note

All claims expressed in this article are solely those of the authors and do not necessarily represent those of their affiliated organizations, or those of the publisher, the editors and the reviewers. Any product that may be evaluated in this article, or claim that may be made by its manufacturer, is not guaranteed or endorsed by the publisher.
